# Comprehensive analysis of pyroptosis-related genes in psoriasis and targeted gene editing of CASP1 and CASP5 using lipid nanoparticles to alleviate skin inflammation

**DOI:** 10.3389/fbioe.2025.1639869

**Published:** 2025-07-23

**Authors:** Gexiao Xu, Guanyi Ma, Jiachen Sun, Xiaoyan Yu, Jie Sun, Bing Gao

**Affiliations:** ^1^ Department of Dermatology, Dermatology Hospital of Xiaoshan District, Hangzhou, China; ^2^ The 988th Hospital of Joint Logistic Support Force of Chinese People’s Liberation Army, Zhengzhou, China; ^3^ Department of Dermatology, Peking University Third Hospital, Beijing, China; ^4^ Department of Burns, Sichuan Academy of Medical Sciences and Sichuan Provincial People’s Hospital, University of Electronic Science and Technology of China, Chengdu, China

**Keywords:** psoriasis, pyroptosis, immune infiltration, risk score, lipid nanoparticles, CRISPR-Cas9

## Abstract

Psoriasis is a chronic inflammatory skin disorder driven by immune dysregulation and excessive cell death. Pyroptosis, a form of inflammatory programmed cell death, has not been extensively studied in the context of psoriasis despite its importance in inflammation. In this study, we systematically analyzed the expression of pyroptosis-related genes (PRGs) in psoriasis to identify critical players involved in disease progression. Using bioinformatics tools and publicly available datasets, we constructed a risk score model based on machine learning algorithms, which identified several key hub genes including CASP1, CASP5, AIM2, GZMB, GZMA, IL1B, and NOD2. The generated risk score model demonstrated robust performance in external validation datasets, showing strong predictive power for psoriasis severity and immune infiltration. High-risk patients exhibited increased inflammatory cell infiltration and worsening clinical symptoms, which was consistent with the model’s ability to predict immune response dynamics in psoriatic lesions. To further validate our findings, we analyzed single-cell RNA sequencing data and demonstrated that the risk score was highly correlated with immune cell composition, particularly DCs, T cells, and mast cells, indicating that patients with higher risk scores have more severe disease and stronger immune infiltration. Additionally, we targeted CASP1 and CASP5 using CRISPR-Cas9 delivery via lipid nanoparticles (LNPs) to selectively knock out these genes in keratinocytes, resulting in significant therapeutic effects in the IMQ-induced psoriasis mouse model. Our findings provide comprehensive insights into the role of pyroptosis in psoriasis and propose a novel gene editing strategy for alleviating the disease.

## Introduction

Psoriasis is a chronic, immune-mediated inflammatory skin disease characterized by excessive keratinocyte proliferation, epidermal hyperplasia, and aberrant immune responses (Raharja et al., 2021; Lowes et al., 2014; Gudjonsson et al., 2004). The condition is marked by persistent lesions and inflammation, often associated with significant morbidity. Although the exact pathogenesis of psoriasis remains incompletely understood, immune dysregulation plays a central role, with T cell activation and cytokine release being critical drivers of disease progression (Li et al., 2020). Type 17 immunity, characterized by the overproduction of IL-17 and IL-23, is considered a hallmark of the disease, further exacerbating skin inflammation and contributing to epidermal hyperplasia (Kagami et al., 2010; Fitch et al., 2007).

Current treatments for psoriasis primarily focus on controlling symptoms and reducing inflammation. These include topical therapies (such as corticosteroids and vitamin D analogs), phototherapy, and systemic treatments (such as immunosuppressive drugs, biologics targeting IL-17, IL-23, and TNF-α) (Trémezaygues and Reichrath, 2011; Dogra and Mahajan, 2013; Roenigk et al., 1998). Although these treatments are effective in managing symptoms for many patients, they have significant limitations. Topical treatments are often only effective for mild-to-moderate cases and can cause skin thinning and other side effects with prolonged use (Murphy and Reich, 2011). Systemic therapies, while effective for severe cases, carry risks of immune suppression and other systemic side effects, and biologics are expensive and often require regular injections (Lebwohl and Ali, 2001). Furthermore, many patients develop tolerance or resistance to these therapies over time, necessitating the need for more targeted and personalized treatments (Heath et al., 2019).

In recent years, the role of programmed cell death in psoriasis has gained increasing attention (Krüger‐Kra et al., 2006). While apoptosis and necrosis have long been implicated in the disease, pyroptosis, a form of inflammatory cell death driven by inflammasomes, has emerged as an important mechanism in immune-mediated skin disorders (Kaštelan et al., 2009; Yao et al., 2024). Pyroptosis is a highly inflammatory process that leads to the release of pro-inflammatory cytokines like IL-1β and IL-18, which are known to contribute to the chronic inflammation observed in psoriasis (Li and Jiang, 2023). However, the exact role of pyroptosis-related genes (PRGs) in psoriasis pathogenesis remains poorly understood, and much of the literature to date has focused on other forms of cell death.

In this study, we aim to investigate the role of pyroptosis-related genes in psoriasis through a comprehensive bioinformatics analysis. Using publicly available datasets, we systematically examined the expression of PRGs in psoriatic lesions and identified key genes involved in disease progression. By constructing a risk score model based on machine learning algorithms, we developed a predictive tool that can assess disease severity and immune infiltration. Furthermore, we used single-cell RNA sequencing to validate the robustness of our findings at the cellular level, and to explore the immune microenvironment of psoriatic skin.

Building on these insights, we sought to test the therapeutic potential of targeting key pyroptosis-related genes, specifically CASP1 and CASP5, in the treatment of psoriasis. We employed CRISPR-Cas9 gene editing, delivered via lipid nanoparticles (LNPs), to selectively knock out Casp1 and Casp11 (murine homolog of human CASP5) in keratinocytes within the IMQ-induced psoriasis mouse model. Our results demonstrate that targeting these key inflammasome components can significantly reduce disease severity, immune cell infiltration, and skin hyperplasia, providing a novel therapeutic strategy for managing chronic inflammatory diseases like psoriasis.

## Materials and methods

### Data collection and preprocessing

The gene expression data for psoriasis were obtained from publicly available datasets, including GSE30999 (Suárez-Fariñas et al., 2012) and GSE13355 (Nair et al., 2009), through the Gene Expression Omnibus (GEO) database. These datasets were preprocessed and normalized using the R package limma (Ritchie et al., 2015). An independent validation dataset was obtained from GEO dataset (GSE41662) and preprocessed using the same normalization procedures.

### Pyroptosis-related genes (PRGs) identification

A curated list of pyroptosis-related genes was compiled from the literature (Zeng et al., 2023). Differentially expressed genes (DEGs) were identified by comparing lesional and normal skin samples, using a |log_2_ fold change| > 1 and adjusted p-value <0.05 as thresholds. The intersection of the DEGs and the PRG list was analyzed.

### Differential gene expression and pathway enrichment analysis

Identification of differentially expressed genes (DEGs) between lesional skin and normal skin samples was performed using DESeq2 (Love et al., 2014). A threshold of |log_2_ fold change| > 1 and adjusted p-value <0.05 was applied to select significant DEGs. To further understand the biological functions and pathways involved, we performed Gene Ontology (GO) and Kyoto Encyclopedia of Genes and Genomes (KEGG) enrichment analysis using the clusterProfiler package (Wu et al., 2021) in R.

### Immune microenvironment characterization

To evaluate the immune landscape associated with pyroptosis activity in psoriasis, we applied the CIBERSORT (Chen et al., 2018) algorithm to deconvolute the immune cell composition from bulk gene expression data. Gene expression matrices from both GSE13355 and GSE30999 datasets were first normalized using the limma and edgeR packages in R. CIBERSORT was then run using the default LM22 immune cell signature matrix, which quantifies 22 distinct human immune cell types, including T cell subsets, dendritic cells (DCs), macrophages, mast cells, and others. We set the number of permutations to 1,000 and filtered output to retain samples with CIBERSORT p-values <0.05, indicating statistically reliable estimates. Immune cell fractions were compared between high- and low-risk score groups using the Wilcoxon rank-sum test, and the relationship between individual immune cell types and risk score was evaluated using Spearman correlation. The immune infiltration profiles were visualized using heatmaps and violin plots generated with ggplot2 and ComplexHeatmap packages.

### Risk score model construction

To develop a pyroptosis-related risk score model for psoriasis, we began by identifying differentially expressed pyroptosis-related genes (PRGs) from lesional versus non-lesional skin using the GSE13355 dataset. DEGs were selected using a threshold of adjusted p-value <0.05 and |log2 fold change| > 1, and were then intersected with a curated list of 46 PRGs derived from previous literature. The selected PRGs were subjected to least absolute shrinkage and selection operator (LASSO) regression using the glmnet package in R. Ten-fold cross-validation was employed to select the optimal value of the regularization parameter lambda that minimized prediction error. Patients were then stratified into high- and low-risk groups based on the median risk score. The model’s predictive performance was validated in the independent dataset, using Receiver Operating Characteristic (ROC) curve analysis with the pROC package. Additionally, we evaluated the correlation between the risk score and immune infiltration patterns (from CIBERSORT) to assess biological relevance.

### Single-cell RNA sequencing analysis

Single-cell RNA-seq data of psoriatic skin samples were downloaded from public repositories (GSE228421) (Francis et al., 2024) and processed using the Seurat package (Satija et al., 2015) in R. The data underwent standard preprocessing steps, including quality control, normalization, and scaling. The cells were clustered based on gene expression profiles using graph-based clustering. Immune cell subtypes were identified using known marker genes. The relationship between risk score and immune cell composition was examined by comparing the distribution of cell types in high- and low-risk groups. Uniform manifold approximation and projection (UMAP) was used for visualization of immune cell clusters. Cell-cell communication networks were inferred using CellChat (Jin et al., 2021), and interaction strengths were compared between risk groups.

### Animal model and LNP-mediated CRISPR-Cas9 delivery

Female C57BL/6 mice (8 weeks old) were used for the IMQ-induced psoriasis model. Mice were treated topically with 5% imiquimod (IMQ) cream on their ears for 8 consecutive days to induce psoriasis-like skin inflammation. The control group was treated with vehicle (petrolatum). Lipid nanoparticles (LNPs) were prepared using a standard method with DLin-MC3-DMA as the lipid component (Ermilova and Swenson, 2020). LNPs were loaded with gRNAs targeting Casp1 and Casp11 and delivered topically to the ears of IMQ-treated mice on days 1, 3, 5, and 7 of the treatment regimen. The gRNA sequences targetting Casp1 and Casp11 were described before (Yu et al., 2024). Ear thickness was measured using a digital micrometer at the indicated time points (days 0, 2, 4, 6, 8). PASI scores were assessed based on erythema, scaling, and thickness using a previously established scoring system (Li et al., 2021). Ears were harvested at the end of the treatment and fixed in 10% formalin. Tissues were embedded in paraffin, sectioned, and stained with H&E for histopathological examination. The C57BL/6J mice were obtained from The Jackson Laboratory. Animal care, feeding, housing, and enrichment were accomplished in the Shang Hai MODEL ORGANISMS. All the surgeries on the mice were carried out under isoflurane anesthesia and carbon dioxide euthanasia was used all the time to sacrifice the mice. 3 mice of 8 week old were included in each group. All animal-related experiments were reviewed and approved by the Shanghai Model Organism Ethics Committee.

### Statistical analysis

All statistical analyses were conducted using R (v4.1.2) and Python (v3.8). Survival Data are presented as mean ± SD. Statistical significance was determined using one-way ANOVA followed by Tukey’s *post hoc* test for comparisons between multiple groups. p-values <0.05 were considered statistically significant.

## Results

### Identification and characterization of pyroptosis-related genes (PRGs) in psoriasis

**FIGURE 1:**
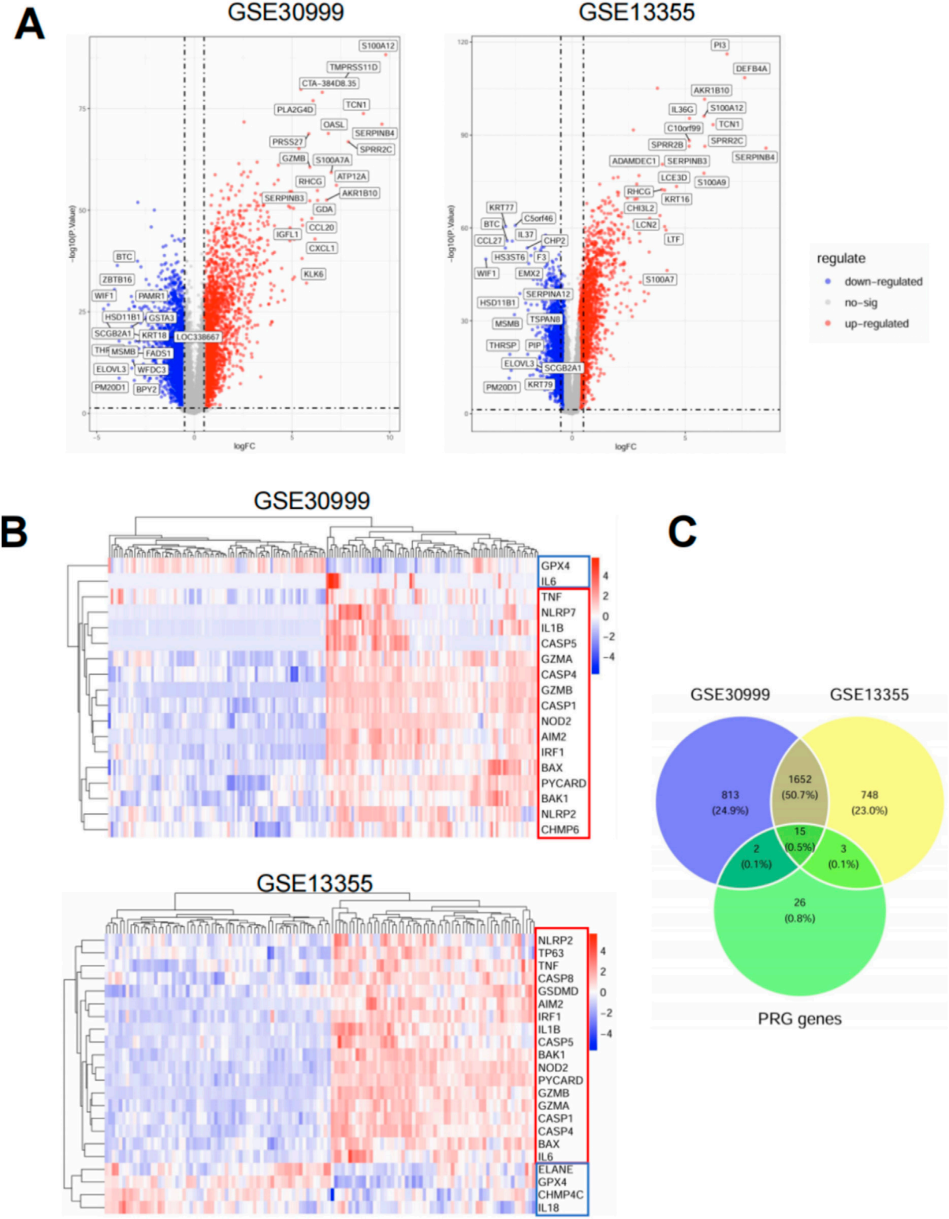
Identification and characterization of differentially expressed pyroptosis-related genes (PRGs) in psoriasis. **(A)** Volcano plots displaying differentially expressed genes (DEGs) between psoriatic lesional skin and normal skin in two publicly available microarray datasets: GSE30999 (left) and GSE13355 (right). Red dots represent upregulated genes, blue dots represent downregulated genes, and gray dots indicate genes without significant changes. DEGs were defined using the cutoff of |log_2_ fold change| > 1 and adjusted *p* < 0.05. Selected PRGs are labeled on the plots. **(B)** Heatmaps of PRG expression in GSE30999 (top) and GSE13355 (bottom). Data were normalized and Z-scored by gene across samples. Rows represent individual PRGs and columns represent patient samples. Hierarchical clustering was applied to both genes and samples using Euclidean distance and complete linkage. Red and blue color scales indicate high and low expression, respectively. **(C)** Venn diagram showing the overlap of DEGs between the two datasets and the curated list of 46 pyroptosis-related genes. A total of 1,652 DEGs were shared between GSE30999 and GSE13355. Among them, 15 genes overlapped with the PRG list and were consistently dysregulated in both datasets.

To explore the role of pyroptosis in psoriasis, we performed differential expression analysis on two independent microarray datasets (GSE30999 and GSE13355). As shown in the volcano plots (Figure 1A), both datasets revealed widespread transcriptional changes between psoriatic and healthy skin, with a large number of significantly upregulated genes. Among 46 curated pyroptosis-related genes (PRGs), 20 were differentially expressed in at least one dataset, and 15 overlapped between both datasets (Figure 1C). These shared PRGs—such as IL1B, CASP1, TNF, GSDMD, and AIM2—were predominantly upregulated and are known to participate in key inflammatory and cell death pathways. Heatmap clustering based on PRG expression profiles clearly separated lesional from normal samples in both datasets (Figure 1B), Most PRGs were upregulated in psoriasis samples. highlighting their diagnostic and biological relevance. Overall, these findings indicate that pyroptosis-associated gene dysregulation is a reproducible feature of psoriatic lesions, suggesting a potential role for pyroptosis in disease pathogenesis.

### Functional enrichment reveals immune activation and metabolic suppression in psoriatic lesions

**FIGURE 2:**
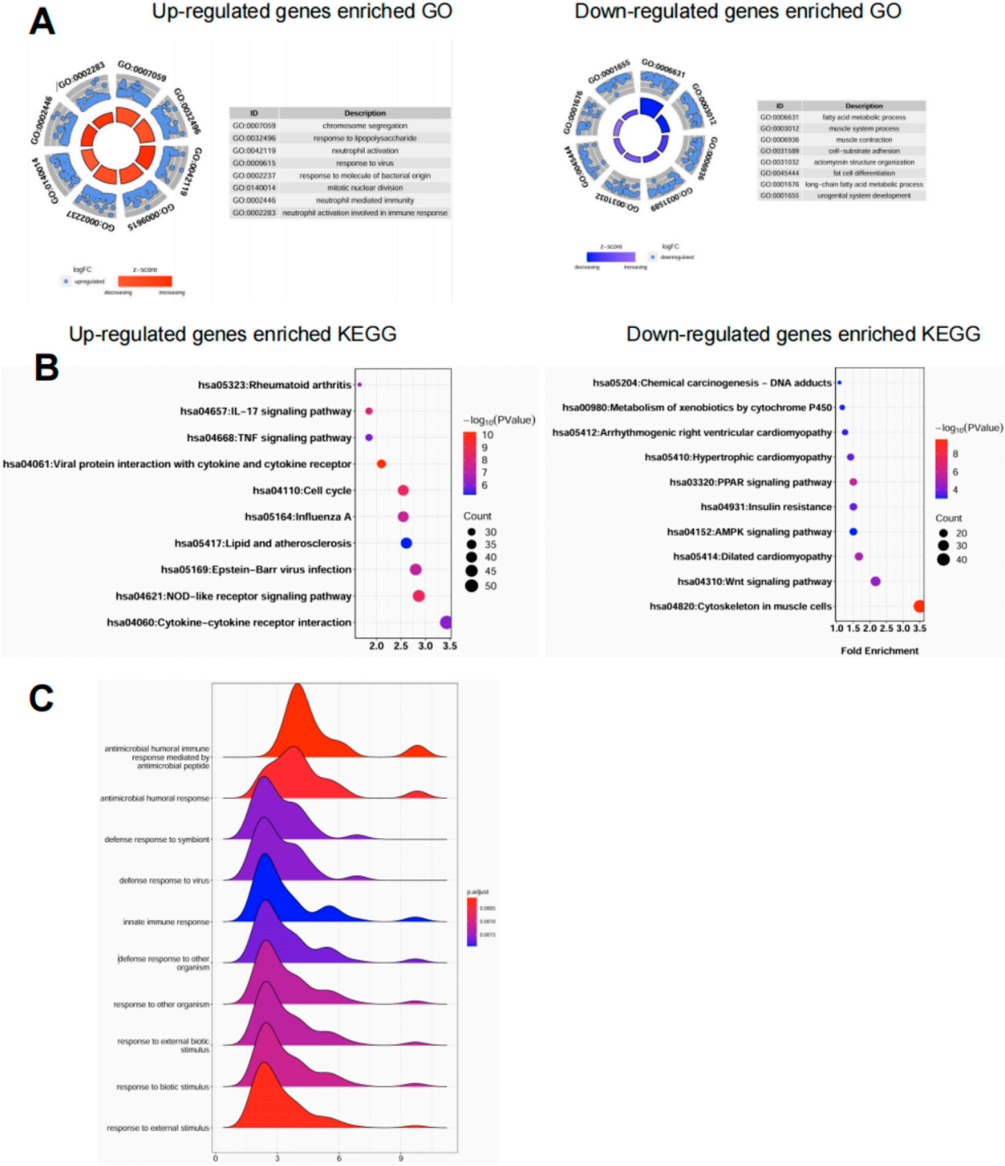
Functional enrichment reveals immune activation and metabolic suppression in psoriatic lesions. **(A)** GO enrichment analysis of upregulated (left) and downregulated (right) genes from GSE30999 and GSE13355. Top enriched biological processes were visualized using circular barplots. Upregulated genes were mainly associated with immune activation, while downregulated genes were enriched in lipid metabolism and epidermal development. **(B)** KEGG pathway enrichment analysis showing the top 10 pathways for upregulated (left) and downregulated (right) genes. Upregulated pathways included IL-17, TNF, and NOD-like receptor signaling, while downregulated pathways were related to metabolism and structural maintenance. **(C)** GSEA ridge plots highlighting immune-related GO terms enriched in psoriatic lesions. Curves represent the distribution of genes ranked by expression, with color indicating adjusted *p*-value. Most immune pathways showed positive enrichment in lesional samples across both datasets.

To investigate the biological significance of differentially expressed genes (DEGs), we performed GO and KEGG enrichment analyses using the DEGs shared by both GSE30999 and GSE13355 datasets. GO analysis showed that upregulated genes were predominantly enriched in immune-related biological processes such as neutrophil activation, cytokine-mediated signaling, and regulation of innate immune response (Figure 2A, left). In contrast, downregulated genes were associated with epidermal differentiation, fatty acid metabolism, and skin barrier development (Figure 2A, right). KEGG pathway analysis revealed that upregulated genes were significantly involved in inflammatory and immune signaling pathways, including IL-17 signaling, TNF signaling, NOD-like receptor signaling, and cytokine–cytokine receptor interaction (Figure 2B, left). These pathways are well-known to be dysregulated in psoriasis and are consistent with its inflammatory nature. Conversely, downregulated genes were enriched in metabolic and structural pathways such as PPAR signaling, AMPK signaling, Wnt signaling, and cytoskeletal regulation (Figure 2B, right), indicating impaired metabolic and differentiation programs in psoriatic skin. GSEA analysis further demonstrated consistent and significant enrichment across both datasets, with notably low adjusted *p*-values for processes such as antimicrobial humoral responses, innate immune activation, and defense response to pathogens (Figure 2C). Together, these findings support a model in which psoriatic inflammation is accompanied by robust upregulation of innate immune pathways and suppression of homeostatic epidermal programs.

### Pyroptosis-based risk score model distinguishes psoriatic lesions and performs well in external validation

**FIGURE 3:**
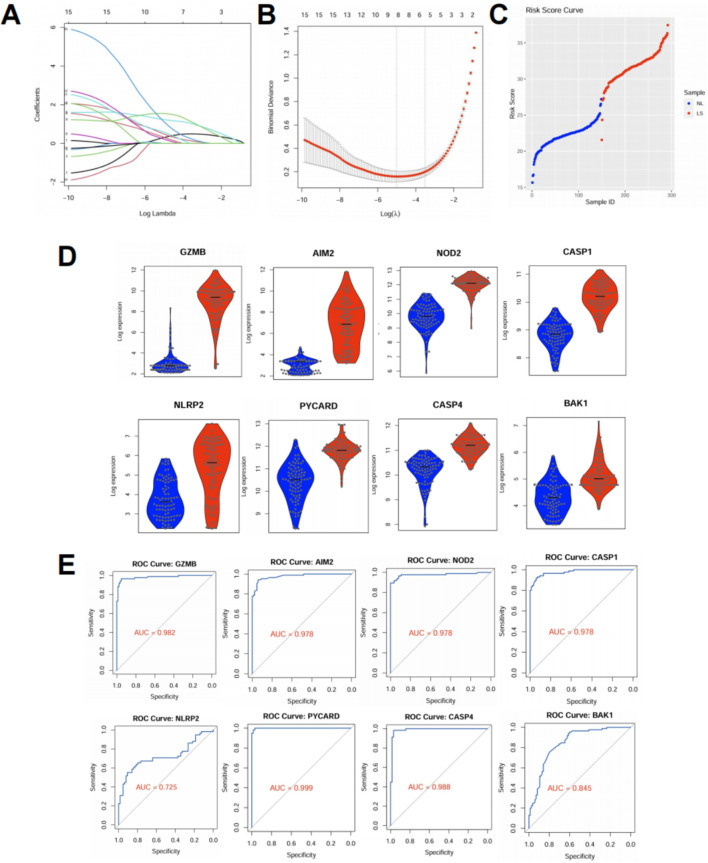
Pyroptosis-based risk score model distinguishes psoriatic lesions and performs well in external validation. **(A,B)** LASSO logistic regression was applied to the training dataset (GSE13355) using expression profiles of differentially expressed pyroptosis-related genes (PRGs). **(A)** Coefficient profiles for candidate genes. **(B)** Ten-fold cross-validation identified the optimal lambda value corresponding to minimum binomial deviance. **(C)** Risk scores were calculated based on the selected PRGs. Lesional skin (LS) samples showed significantly higher risk scores than normal skin (NL), effectively distinguishing the two groups. **(D)** Violin plots showing expression of the selected PRGs in the external validation dataset (GSE41662). All genes were significantly upregulated in psoriatic lesions (*p* < 0.001). **(E)** ROC curves evaluating the diagnostic performance of the selected PRGs in GSE41662. All genes exhibited high area under the curve (AUC) values, indicating strong discriminatory power.

To determine whether pyroptosis-related genes (PRGs) could serve as effective biomarkers for psoriasis, we constructed a diagnostic risk score model using the datasets. First, we performed LASSO logistic regression (Morawska et al., 2024) on the differentially expressed PRGs, and identified 8 genes with non-zero coefficients under the optimal lambda value determined by ten-fold cross-validation (Figures 3A,B). These genes were used to calculate a risk score for each sample, which clearly separated lesional (LS) from normal (NL) skin (Figure 3C). The risk score was calculated as: Risk score = (1.129 × expression of CASP1) + (1.549 × PYCARD) + (0.306 × GZMB) + (0.486 × AIM2) + (0.318 × NLRP2) + (1.230 × NOD2) + (0.580 × CASP4) + (0.224 × BAK1). Lesional skin (LS) samples showed significantly higher risk scores than normal skin (NL). The model demonstrated high discriminative power within the training set. To evaluate the robustness of this model, we tested it in an independent external dataset (GSE41662). All 8 PRGs included in the model—GZMB, AIM2, NOD2, CASP1, NLRP2, PYCARD, CASP4, and BAK1—showed significantly elevated expression in lesional samples compared to normal controls (*p* < 0.001 for all genes, Figure 3D). ROC curve analysis further confirmed the diagnostic value of these genes: most achieved excellent classification performance with area under the curve (AUC) values exceeding 0.95, including PYCARD (AUC = 0.999), CASP4 (AUC = 0.988), and GZMB (AUC = 0.982), while NLRP2 showed moderate predictive value (AUC = 0.725) (Figure 3E). Together, these results demonstrate that a PRG-based signature can robustly distinguish psoriatic lesions from normal skin across independent datasets. The high AUC values indicate that these genes not only contribute to disease pathogenesis but may also serve as reliable molecular markers for diagnostic stratification.

### Upregulated PRGs form a dense interaction network

**FIGURE 4:**
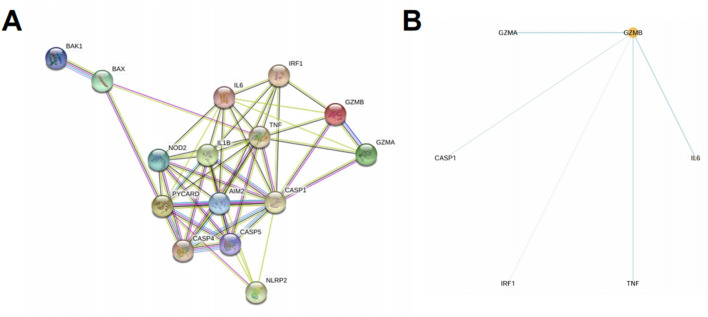
Upregulated PRGs form a dense interaction network centered on GZMB and CASP1. **(A)** PPI network of the 15 upregulated PRGs identified in both GSE30999 and GSE13355, constructed using the STRING database. Edges represent known or predicted protein associations based on experimental data, co-expression, databases, and text mining. **(B)** Hub gene analysis using Cytoscape with the cytoHubba plugin identified GZMB as the central gene based on degree score. Direct interactions of GZMB with other PRGs are shown.

To investigate the interactions among the 15 upregulated pyroptosis-related genes (PRGs) identified in both datasets, we constructed a protein–protein interaction (PPI) network using the STRING database. The resulting network revealed extensive interconnectivity among inflammasome components, caspases, cytokines, and effector molecules (Figure 4A). Notably, GZMB and CASP1 occupied central positions within the network, each displaying multiple direct links with other PRGs, suggesting potential roles as core regulators of pyroptotic signaling in psoriasis. Hub gene analysis using the cytoHubba plugin in Cytoscape further confirmed the centrality of GZMB and CASP1 based on degree score. GZMB was connected to IL6, TNF, CASP1, IRF1, and GZMA, while CASP1 bridged key components of the canonical inflammasome pathway, including AIM2, IL1B, and PYCARD (Figure 4B). These findings imply that GZMB and CASP1 may jointly orchestrate inflammation and cell death processes in keratinocytes during psoriasis progression.

### High pyroptosis risk score correlates with increased infiltration of inflammatory immune cells

**FIGURE 5:**
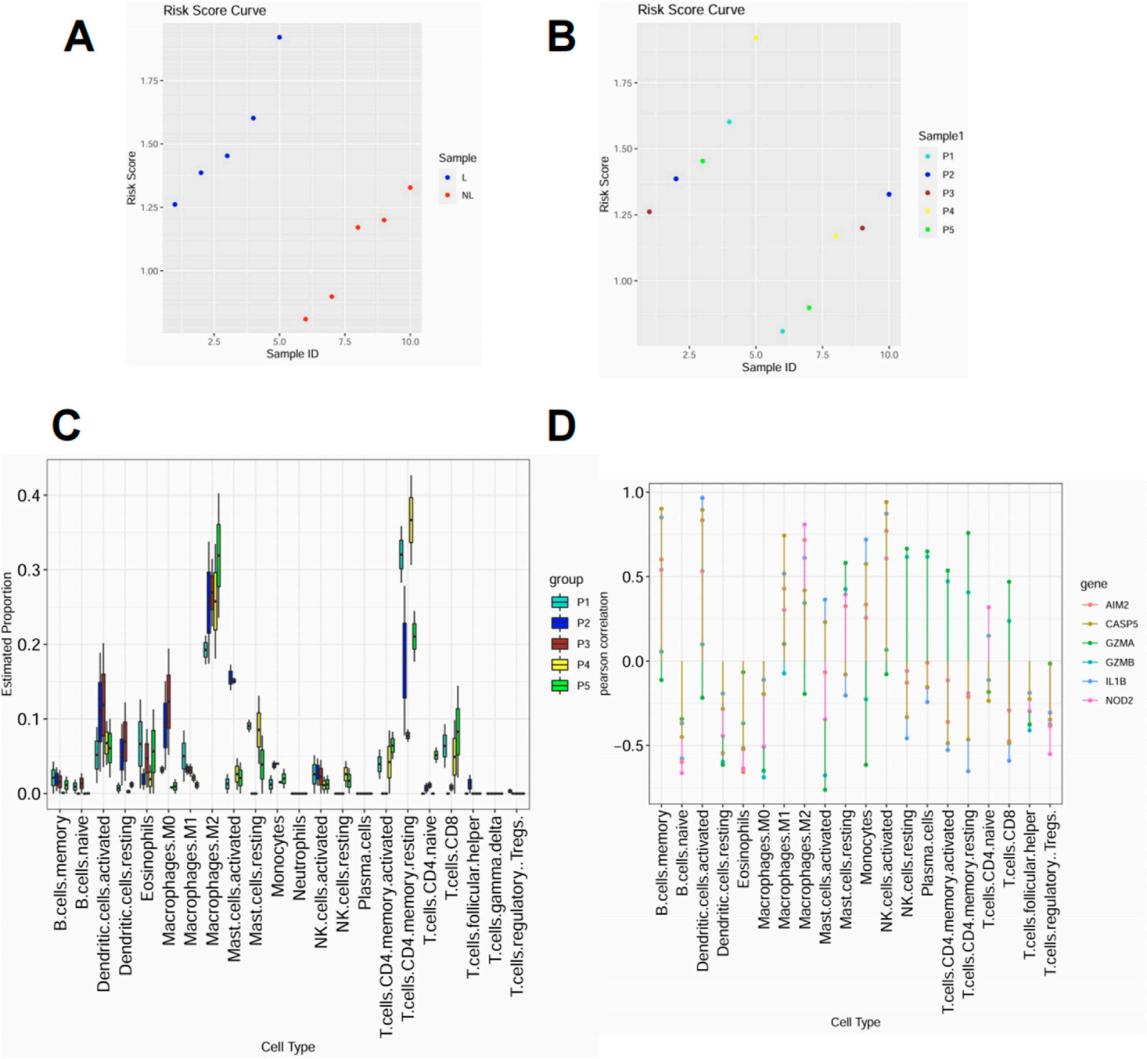
High pyroptosis risk score correlates with increased infiltration of inflammatory immune cells **(A)** Risk score distribution in lesional (L) and normal (NL) skin samples. Lesional samples showed consistently higher scores. **(B)** Samples were stratified into five groups (P1–P5) according to increasing risk scores. **(C)** Immune cell composition was inferred using CIBERSORT. Inflammatory cell types including activated dendritic cells, neutrophils, and M1 macrophages were more abundant in high-risk groups (P4–P5). **(D)** Spearman correlation between selected PRGs and immune cell types. Proinflammatory genes such as GZMB, IL1B, and CASP5 showed positive correlations with inflammatory immune subsets.

To explore the immunological landscape associated with pyroptosis activation, we applied our risk score model to a subset of psoriasis patients and observed significantly elevated scores in lesional skin compared to normal controls (Figure 5A). Patients were divided into five groups (P1–P5) based on ascending risk scores (Figure 5B), representing increasing pyroptotic level. Immune cell profiling using CIBERSORT revealed that patients in high-risk groups (P4 and P5) exhibited greater infiltration of proinflammatory cell types, including activated dendritic cells, mast cells, CD4 T cells and M2 macrophages (Figure 5C). This trend suggests a link between pyroptosis activation and local immune dysregulation. Correlation analysis showed that several key PRGs—GZMB, CASP5, IL1B, AIM2, and NOD2—were positively associated with these inflammatory populations (Figure 5D), further implicating pyroptosis in shaping the immune microenvironment of psoriatic lesions.

### High pyroptosis risk score is associated with increased infiltration of inflammatory immune cells in skin tissue at the single-cell level

To further investigate the cellular composition associated with pyroptosis activation in psoriasis, we analyzed a publicly available single-cell RNA-seq dataset of psoriatic skin. Patients were stratified into high-risk and low-risk groups based on their bulk-tissue pyroptosis risk scores, and single-cell data were visualized by UMAP.

**FIGURE 6:**
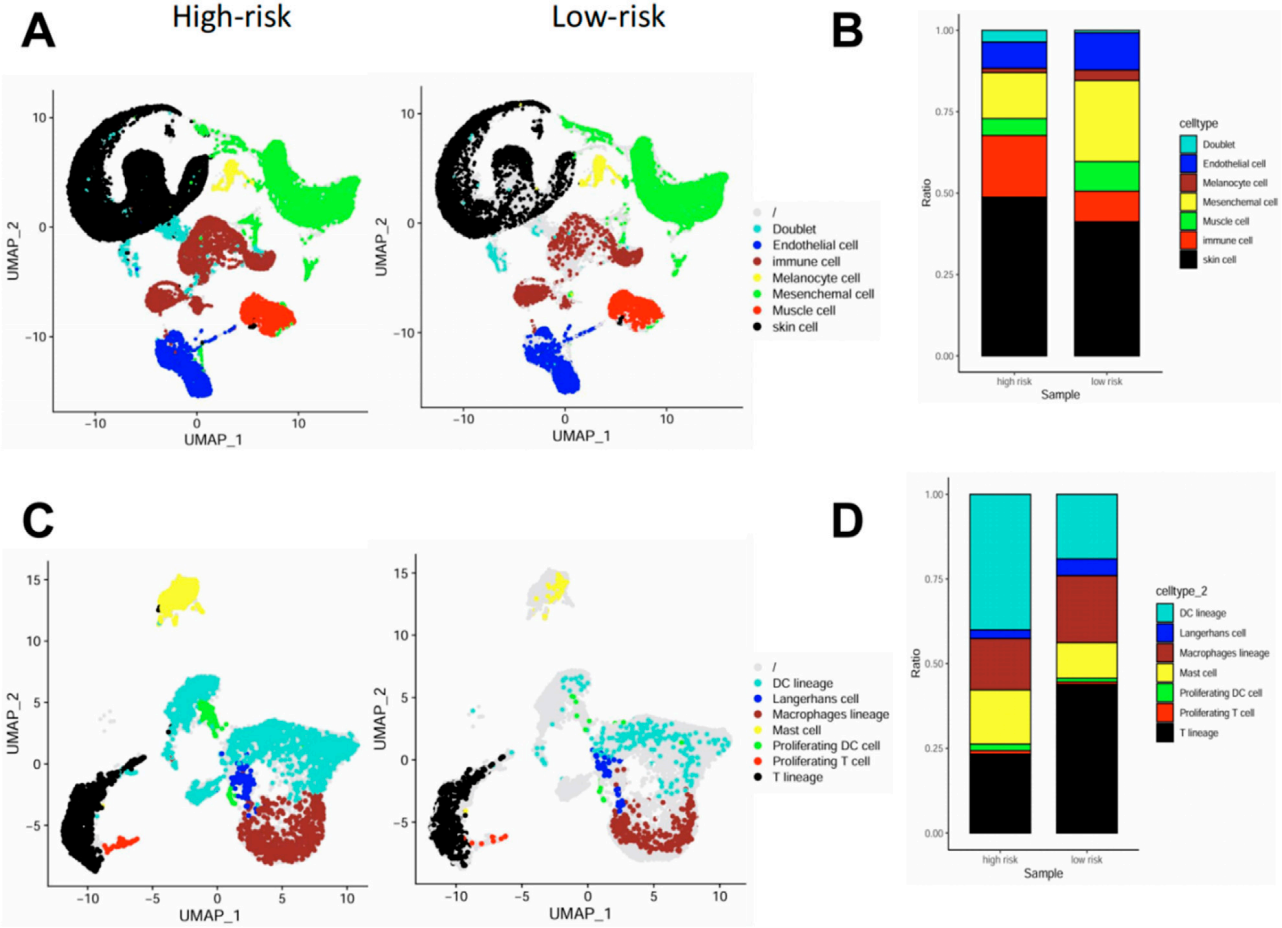
High pyroptosis risk score is associated with increased infiltration of inflammatory immune cells in skin tissue at the single-cell level. **(A,B)** UMAP visualization **(A)** and cell composition analysis **(B)** of major cell types in high-vs. low-risk psoriatic skin samples, showing increased immune, mesenchymal, and endothelial cell fractions in high-risk lesions. **(C,D)** UMAP **(C)** and barplot **(D)** of sub-clustered immune cell populations. High-risk lesions showed increased infiltration of DC lineage cells, macrophages, and proliferating immune cells, while low-risk lesions had higher proportions of Langerhans cells and mast cells.

At the broad cell type level, high-risk samples showed a higher proportion of skin structural cells (e.g., keratinocytes) and an increased proportion of immune cells compared to low-risk samples (Figures 6A,B). These reflected the two basic features of skin chronic inflammation: Epidermal hyperplasia and immune infiltration. To dissect the immune compartment in greater detail, we performed subclustering of immune cells. UMAP and composition analysis revealed that high-risk lesions were enriched for DCs, T cells and mast cells, while low-risk lesions were relatively enriched in macrophages (Figures 6C,D). These findings suggest that pyroptosis activation correlates with heightened immune cell infiltration and expansion in the psoriatic skin microenvironment, supporting the proinflammatory nature of the high-risk group.

### High pyroptosis risk score is associated with enhanced immune cell–cell communication in psoriatic skin

**FIGURE 7:**
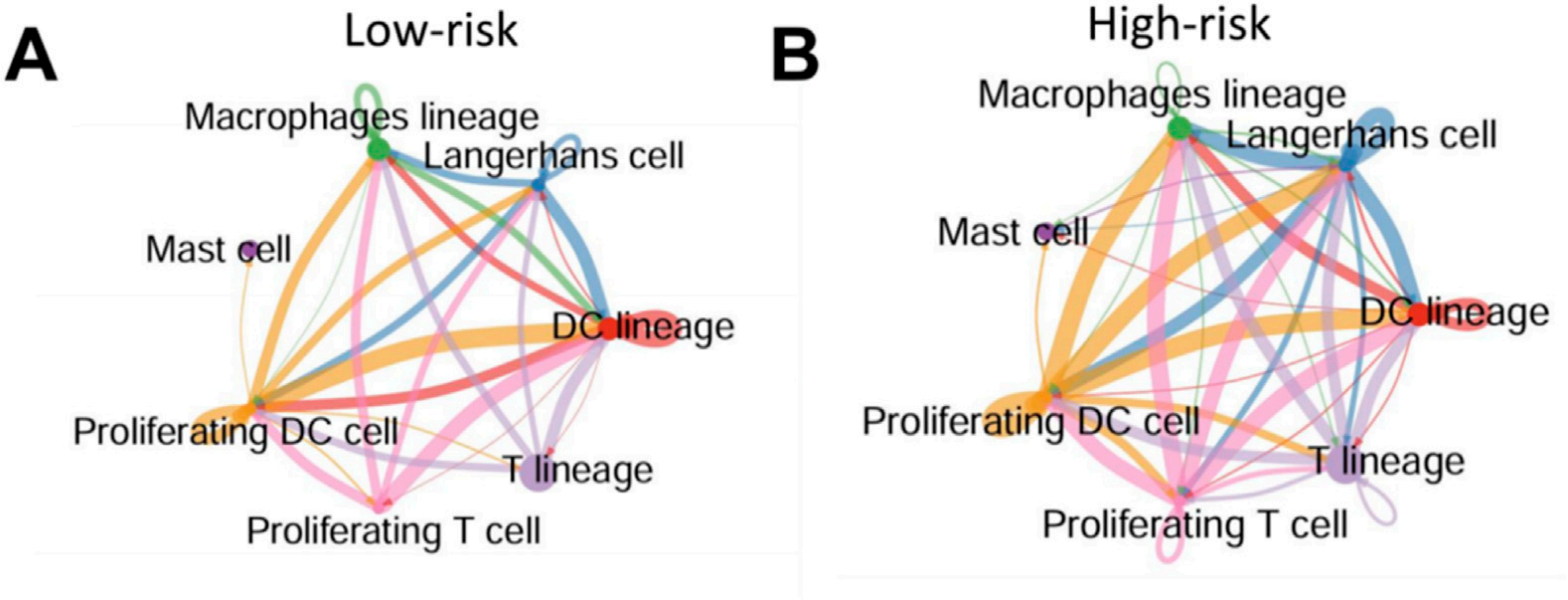
High pyroptosis risk score is associated with enhanced immune cell–cell communication in psoriatic skin **(A,B)** Predicted intercellular communication networks among immune cell subsets in low-risk **(A)** and high-risk **(B)** psoriatic skin. Edges represent predicted ligand–receptor interactions between cell types; edge width reflects interaction strength. High-risk samples displayed more extensive and stronger immune cell connectivity, particularly involving proliferating T cells, DCs, and mast cells.

To explore how pyroptosis activation influences immune cell interactions in the psoriatic microenvironment, we inferred intercellular communication networks among immune subsets based on known ligand-receptor pairs. In low-risk patients, cell-cell interactions were relatively sparse and concentrated around DC lineage, macrophages, and T cells (Figure 7A). In contrast, high-risk patients exhibited a much more interconnected immune network, with intensified interactions particularly involving proliferating T cells, proliferating DCs, and mast cells (Figure 7B).

These results suggest that pyroptosis activation is not only associated with immune infiltration, but may also amplify immune cell crosstalk, potentially exacerbating local inflammation and epidermal damage.

### Targeted delivery of CRISPR-Cas9 to keratinocytes alleviates psoriasis-like skin inflammation *in vivo*

**FIGURE 8:**
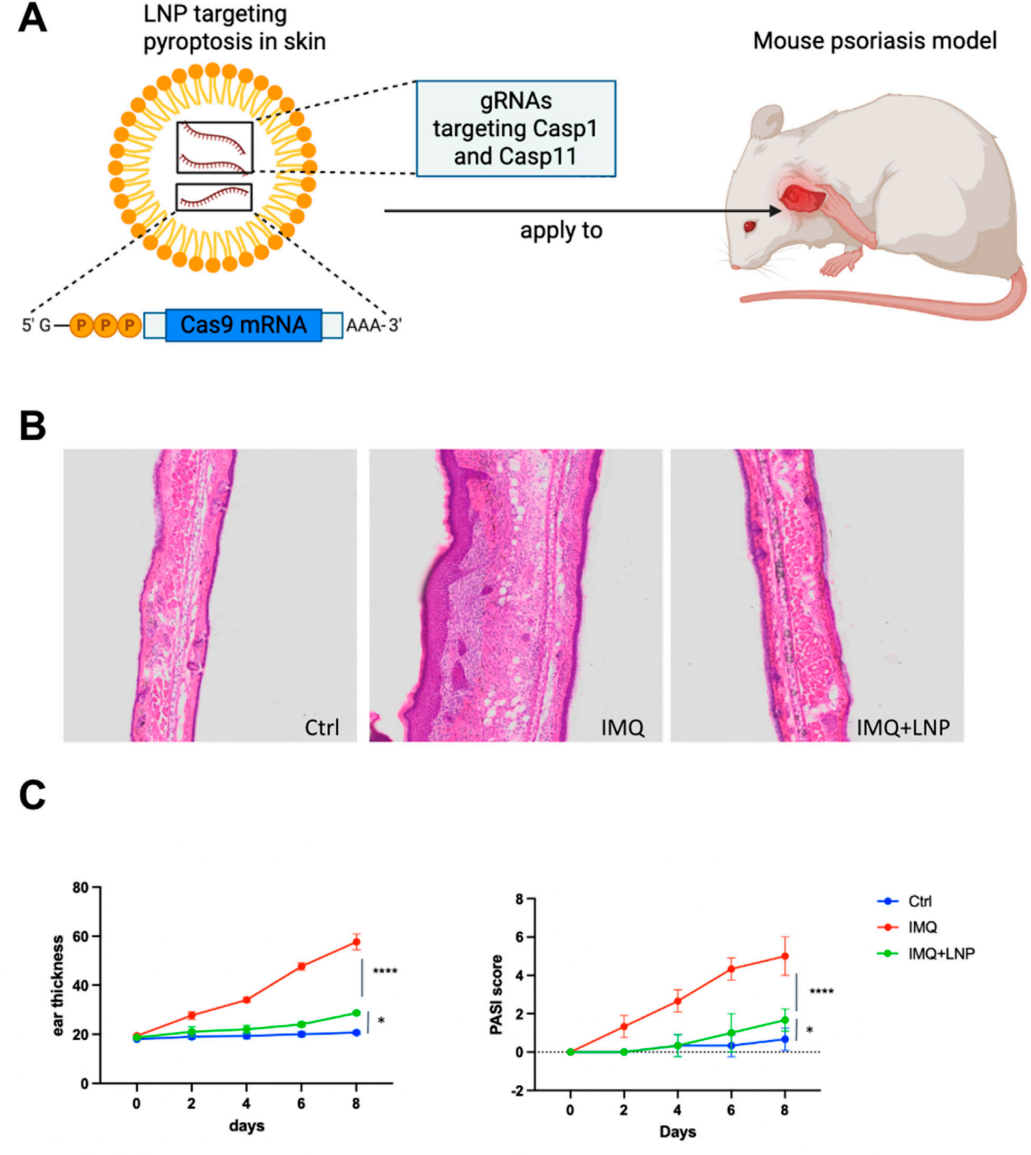
Targeting pyroptosis in skin using LNP-delivered CRISPR-Cas9 to alleviate psoriasis in a mouse model **(A)** Schematic illustration of LNP-mediated CRISPR-Cas9 delivery targeting pyroptosis in skin. LNPs are loaded with gRNAs targeting Casp1 and Casp11 to knockout these genes in keratinocytes in the psoriatic mouse model. The LNP formulation is applied topically to the skin. **(B)** Representative H&E stained sections of mouse ear tissues from the three experimental groups: Ctrl, IMQ, and IMQ + LNP-Cas1/11 gRNA. Control group shows normal skin architecture, IMQ treatment results in epidermal hyperplasia and inflammatory cell infiltration, while the LNP + IMQ group shows a reduction in both epidermal thickness and immune cell infiltration after Casp1/11 knockout. **(C)** Ear thickness (left) and PASI score (right) measurements over the course of 8 days. The IMQ group shows significant thickening and increased PASI score, while the IMQ + LNP-Cas1/11 gRNA group exhibits reduced ear thickness and PASI scores compared to the IMQ group, indicating the therapeutic potential of Casp1/11 knockout. Data represent mean ± SEM, with statistical significance indicated by *p* values (****: *p* < 0.0001, *: *p* < 0.05).

To explore the therapeutic potential of targeting these hub genes in alleviating psoriasis, we used Lipid Nanoparticles (LNPs) to deliver gRNAs targeting Casp1 and Casp5 to keratinocytes in the IMQ-induced psoriasis mouse model (Figure 8A). Casp1 and Casp5 were selected as targets for gene knockout because they are key players in the pyroptosis pathway and also enriched in our hub gene analysis. Also, AIM2 and NOD2 are key players in pyroptosis (hub genes) identified through our analysis in psoriasis. Both of these genes play significant roles in activating the inflammasome, with CASP1 being a common downstream effector. Upon activation, both AIM2 and NOD2 trigger the formation of inflammasomes that ultimately lead to CASP1 activation, which processes pro-inflammatory cytokines such as IL-1β, which is also a key player in psoriasis through our former analysis. Given this critical role of CASP1 in amplifying inflammation, and the fact that CASP5 is similarly involved in downstream pyroptotic signaling, targeting CASP1 and CASP5 emerges as the most effective strategy to mitigate the inflammatory response in psoriasis. So these genes were selected because they are critical upstream regulators of pyroptosis, making them more likely to have a broad therapeutic impact compared to targeting downstream effectors like GZMB.

LNPs (lipid nanoparticles) were chosen as the delivery vehicle due to their ability to efficiently encapsulate and deliver CRISPR-Cas9 components to keratinocytes both in animal model and human (Zeng et al., 2025; Bolsoni et al., 2023; Lee et al., 2025). Keratinocytes are the primary cell type involved in psoriasis pathogenesis, and they turnover rapidly, minimizing the risk of off-target mutations in long-lived cells. Topical delivery of LNPs to the skin allows for targeted delivery to epidermal keratinocytes, ensuring high local concentrations of CRISPR-Cas9 components without significant systemic exposure.

Histological analysis of H&E-stained skin sections revealed that IMQ treatment caused significant epidermal hyperplasia and inflammatory cell infiltration (Figure 8B, middle). In contrast, the IMQ + LNP group showed reduced epidermal thickness and fewer infiltrating immune cells, indicating that targeting Casp1 and Casp11 (murine homolog of CASP5) successfully mitigated the inflammatory response in the skin (Figure 8B, right). The control group exhibited normal skin structure with no noticeable inflammation (Figure 8B, left).

Quantitative measurements of ear thickness (Figure 8C, left) and PASI scores (Li et al., 2021) (Figure 8C, right) confirmed these histological findings. The IMQ group showed progressive increases in both ear thickness and PASI scores, consistent with the severe inflammation associated with psoriasis. In contrast, the IMQ + LNP group exhibited significantly lower ear thickness and PASI scores, indicating a marked reduction in disease severity. The control group showed no change over time, with consistent baseline values.

These results demonstrate that CRISPR-Cas9-mediated knockout of Casp1 and Casp11 in keratinocytes, using LNPs for targeted delivery, effectively reduces the inflammatory response and mitigates psoriasis symptoms in the mouse psoriasis model, highlighting the potential of this strategy as a novel therapeutic approach for inflammatory skin diseases.

## Discussion

In this study, we systematically investigated the role of pyroptosis-related genes (PRGs) in psoriasis and developed a machine learning-based risk score model that accurately predicts disease severity and immune infiltration. Our analysis identified CASP1, CASP5, AIM2, NOD2, GZMB, GZMA, and IL1B as key hub genes in the inflammatory pathways driving psoriasis pathogenesis. Notably, CASP1 and CASP5 were highlighted as central downstream effectors of pyroptosis, making them attractive therapeutic targets for modulating skin inflammation and hyperplasia in psoriasis.

Our risk score model, based on the expression of these PRGs, demonstrated robust predictive power in external validation datasets, effectively distinguishing high-risk from low-risk patients. High-risk patients, as indicated by the risk score, exhibited significantly more immune cell infiltration and worsening disease severity, consistent with the characteristic type 17 immunity observed in psoriasis. These findings underscore the potential of pyroptosis as a key driver of inflammation in psoriasis, offering new insights into the molecular mechanisms underlying the disease.

Single-cell RNA sequencing further validated the robustness of our risk score model by revealing a strong correlation between immune cell composition and the risk score. High-risk patients showed an enriched population of activated dendritic cells (DCs), T cells, and mast cells, while macrophages were more prominent in low-risk lesions. This finding aligns with previous studies showing that DCs and T cells play a pivotal role in psoriasis inflammation, while macrophages are more involved in the tissue repair process. The immune microenvironment of psoriatic lesions, characterized by heightened immune infiltration, appears to be regulated by pyroptosis and inflammasome activation, making these pathways critical therapeutic targets.

One of the most significant findings of this study is the successful application of LNP-mediated CRISPR-Cas9 gene editing to target CASP1 and CASP5 in keratinocytes. Both CASP1 and CASP5 can independently induce pyroptosis, and since both genes were identified as key regulators in our analysis, we chose to target both to minimize functional redundancy. By selectively knocking out these genes, we were able to significantly reduce the severity of psoriasis in the IMQ-induced mouse model, as evidenced by decreased ear thickness, lower PASI scores, and reduced immune cell infiltration. CASP1 has long been recognized as a central mediator of IL-1β and IL-18 processing, two cytokines that contribute to the chronic inflammation in psoriasis. CASP5, although less studied, appears to play a similar role in regulating inflammasome-induced pyroptosis, and our results suggest that targeting both CASP1 and CASP5 may offer a more comprehensive approach to mitigating inflammation.

The use of LNPs for targeted delivery of CRISPR-Cas9 components to keratinocytes offers a promising, non-invasive method for gene editing in skin diseases. The topical application of LNPs ensures high local concentrations of the CRISPR system without systemic exposure, minimizing potential off-target effects. Compared to existing topical agents such as corticosteroids or vitamin D analogs, our approach offers the advantage of targeting upstream inflammatory machinery (e.g., inflammasome components) rather than downstream cytokines. Furthermore, unlike biologics that require repeated systemic administration and may cause immunosuppression or loss of response over time, CRISPR-Cas9-mediated knockout via LNPs is localized, durable, and potentially administered at much lower frequency. This strategy represents a significant step forward in the development of gene therapy for inflammatory skin diseases, offering a novel method for modulating cell death pathways and restoring immune homeostasis in psoriasis.

While our study provides compelling evidence for the role of pyroptosis in psoriasis and the potential of CASP1 and CASP5 as therapeutic targets, several questions remain to be addressed. Although we have taken measures to ensure the specificity of our gRNAs and minimize off-target effects, the possibility of off-target cleavage remains a concern. Methods such as next-generation sequencing (NGS) can be employed to assess editing fidelity and identify any unintended genetic modifications in the target tissue. Additionally, immune reactions to both the LNP carrier and the Cas9 protein may arise (Chew, 2018), potentially limiting the safety and efficacy of this approach. Future studies should explore the long-term effects of CRISPR-mediated gene editing in psoriasis models, particularly with regard to immune tolerance and off-target effects (Han et al., 2020). In addition, although the gRNAs used for Casp1 and Casp11 knockout were carefully designed for high specificity, potential off-target effects remain a consideration for translational applications. To mitigate this, future studies may incorporate strategies such as high-fidelity Cas9 variants, optimized gRNA design algorithms, and LNP formulations with enhanced tissue and cell-type specificity to further improve safety and precision. Additionally, the broader applicability of our LNP-based CRISPR delivery system to other chronic inflammatory diseases and autoimmune conditions should be investigated.

While our findings in the IMQ mouse model are promising, translating these results to human clinical applications requires several key steps. First, we plan to transition from the murine model to humanized skin models, such as human epidermis-on-a-chip or skin grafts, to more closely simulate the human immune response and skin physiology. These models can be treated with LNP-mediated CRISPR-Cas9 gene editing systems to assess delivery efficiency, editing fidelity, and immune responses in a human context. Another promising approach involves the use of *ex vivo* human skin organoids, which offer a robust system for testing the effects of gene editing on human skin inflammation, cell turnover, and immune modulation. These advanced models will provide critical insights into the safety, efficacy, and long-term effects of our CRISPR-based therapy in human skin, paving the way for clinical trials and therapeutic application in psoriasis and other chronic inflammatory diseases.

Recent studies have increasingly highlighted the involvement of regulated cell death pathways in psoriasis beyond classical apoptosis. For instance, necroptosis and ferroptosis have both been implicated in keratinocyte dysfunction and immune activation in psoriatic lesions, suggesting that multiple forms of cell death may interact to shape disease pathogenesis (Duan et al., 2020; Shou et al., 2021). Moreover, the therapeutic potential of modulating inflammasome pathways has gained traction, with several studies exploring NLRP3 inhibitors or IL-1β blockade in preclinical models of psoriasis and other chronic inflammatory diseases (Tsuji et al., 2020). Our work contributes to this growing field by integrating bioinformatics-guided target discovery with precise, keratinocyte-focused genome editing, offering a proof-of-concept for rationally designed, cell-type-specific interventions in skin inflammation.

In conclusion, our study provides new insights into the molecular mechanisms of psoriasis by identifying key pyroptosis-related genes and demonstrating the potential of targeted gene editing as a therapeutic approach. The CRISPR-Cas9/LNP strategy represents a promising tool for precision medicine, offering a targeted approach to modulating the immune response and cell death pathways in chronic inflammatory diseases like psoriasis.

## Conclusion

In this study, we provide a comprehensive analysis of pyroptosis-related genes (PRGs) in psoriasis, identifying key molecular players such as CASP1, CASP5, AIM2, NOD2, and IL1B that drive the inflammatory response and disease progression. Our findings underscore the critical role of pyroptosis in psoriasis pathogenesis and demonstrate that targeting CASP1 and CASP5 through CRISPR-Cas9 gene editing can effectively alleviate disease symptoms and reduce inflammation in the IMQ-induced mouse model. By constructing a machine learning-based risk score model and validating it across external datasets, we established a robust tool for predicting disease severity and immune infiltration in psoriasis. Our single-cell RNA sequencing analysis further validated the model, highlighting the strong association between high-risk scores and increased immune cell infiltration, specifically DCs, T cells, and mast cells. The use of lipid nanoparticles (LNPs) for targeted CRISPR delivery to keratinocytes represents a promising therapeutic strategy for psoriasis and other chronic inflammatory skin diseases. This approach offers a non-invasive, highly localized method for gene editing, minimizing systemic effects and enhancing therapeutic efficacy. Taken together, our findings offer new insights into the role of pyroptosis in psoriasis and pave the way for gene-based therapies targeting key inflammasome components, providing a foundation for the development of novel treatments for inflammatory diseases.

## Data Availability

The original contributions presented in the study are included in the article/supplementary material, further inquiries can be directed to the corresponding author.
